# Freeze–Thaw Effect on Road Concrete Containing Blast Furnace Slag: NMR Relaxometry Investigations

**DOI:** 10.3390/ma14123288

**Published:** 2021-06-14

**Authors:** Liliana Maria Nicula, Ofelia Corbu, Ioan Ardelean, Andrei Victor Sandu, Mihai Iliescu, Dorina Simedru

**Affiliations:** 1 Faculty of Civil Engineering, Technical University of Cluj-Napoca, 28 Memorandumului Street, 400114 Cluj-Napoca, Romania; Liliana.nicula@infra.utcluj.ro (L.M.N.); mihai.iliescu@staff.utcluj.ro (M.I.); 2 Research Institute for Construction Equipment and Technology, ICECON S.A. Bucharest, 266 Pantelimon Road, 2nd District, CP 3-33, 021652 Bucharest, Romania; 3 Center of Excellence Geopolymer & Green Technology (CEGeoGTech), School of Materials Engineering, University Malaysia Perlis, Kangar 01000, Malaysia; 4 Department of Physics and Chemistry, Technical University of Cluj-Napoca, 400114 Cluj-Napoca, Romania; ioan.ardelean@phys.utcluj.ro; 5 Faculty of Materials Science and Engineering, “Gheorghe Asachi” Technical University of Iași, 67 Dimitrie Mangeron Street, 700050 Iași, Romania; 6 Romanian Inventors Forum, Sf. P. Movila 3, 700089 Iasi, Romania; 7 INCDO-INOE2000, Subsidiary Research Institute for Analytical Instrumentation, ICIA Cluj-Napoca, 67 Donath, 400293 Cluj-Napoca, Romania; dorina.simedru@icia.ro

**Keywords:** road concrete, porosity, (NMR) relaxometry, freeze–thaw, blast furnace slag

## Abstract

The present work investigates the effect of freeze–thaw cycles on the porosity of three mixtures of road concrete containing blast furnace slag in comparison with two mixtures made with conventional materials. The main technique used in our investigations is nuclear magnetic resonance (NMR) relaxometry. This permitted the extraction of information with respect to the freeze–thaw effect on pore-size distribution, which influences both the mechanical strength and the molecular transport through the material. Moreover, by using this technique, the structure of the air voids was analyzed for the entire pore system in the cement paste and the aggregate particles. The samples under study were first dried in a vacuum oven and then saturated with water or cyclohexane where the distribution of the transverse relaxation times of the protons was recorded. The NMR relaxation measurements were performed on samples extracted from specimens maintained at 300 freeze–thaw cycles and on control samples extracted from specimens kept in water during the freeze–thaw period. Scanning Electron Microscopy (SEM) was used to analyze the microstructure of concrete samples in order to obtain information about the pore sizes and the distance between them. The results from the NMR relaxation measurements were consistent with those obtained by using standard techniques for determining the porosity and the freeze–thaw resistances. The investigations made it possible to establish the optimal composition of blast furnace slag that can be incorporated into road concrete compositions. This non-invasive technique can also complete standard techniques for assessing the porosity and the progress of internal cracks during the freeze–thaw test.

## 1. Introduction

The resistance to freezing is a factor that needs to be considered when designing road concrete structures because the freeze–thaw phenomenon produces deterioration of the internal structure and on the surface layer of the concrete. If the concrete is exposed to a dry environment the structure is not usually affected by the freeze–thaw phenomenon. However, due to the fact that the road concrete is in contact with water, the water is absorbed by the concrete in the structure of the pores and it generates a high internal tension, which leads to internal structure damage and produces irreversible changes of its properties [1,2,3].

The most important factor influencing freeze–thaw resistance is the distribution and dimensions of the pores [1,4]. The free spaces filled with water in the fresh cement paste or the spaces resulting from the reaction of cement particles during the process of hydration of the cement paste represents the fraction of total volume unoccupied by solid matter and is known as porosity. By mixing components, the cement particles are spread in the total volume of materials and, upon contact with water, reaction takes place between the mineral components and the solution forming around the cement particles. The dispersion of cement particles as evenly as possible is improved by the superplasticizers used [5]. These particles are transformed in colloids by dispersing the solid substance in the liquid due to the forces of attraction at the molecular and atomic level, but the water continues to penetrate inside these colloids. The pore size does not change much during the mixing and dormancy stage in which the activity of the chemical compounds slows down, but there is a change in their size that commences during the hardening stage [6]. The hardened cement paste in the cooling and densifying stage consists of hydrates of different compounds in the form of a gel, calcium hydroxide crystals Ca(OH)_2_, and other minor components, such as un-hydrated cement, and free spaces left in the hydration process [7]. Moreover, additional pores may result in the concrete application stage due to improper compaction. The free spaces that have not been filled with hydration products form capillary pores and the free spaces formed in gel as interconnected spaces define the gel pores. These gel pores can be classified into intra-C-S-H and inter-C-S-H pores [8].

The total volume of voids in concrete can occupy between 1% and 10% of the total volume and a porous concrete at the upper limit indicates low strength [7]. The capillary pores form an interconnected system spread irregularly throughout the mass of the paste [7,9], with sizes ranging from 50 nm and up to 5 µm [10]. The dimensions of the gel pores are much smaller than the capillary pores (approximately 1 ÷ 50 nm) and, therefore, are classified as micropores. The gel pores absorb water even at very low values of ambient humidity [7,9] due to the hygroscopic property of the cement paste and the presence of submicroscopic pores. The spaces with larger dimensions of 5 ÷ 10 µm are considered as entrained air [10].

The PN-EN 480-11 standard [11] describes the air-voids system in the hardened concrete by the following parameters: total air content A (%), specific surface α (mm^2^), spacing factor L (mm) and the micropores content A_300_ (%). The spacing factor (L) of the air pores can be determined using the simplified Powers’ model, which assumes that all air bubbles have the same diameter and are distributed in the corners of a cube. Another approach for determining the spacing factor (L) of the air pores is based on the Pilleo concept, which considers both the air voids system in the cement paste and in the aggregate particles; the principles of the method were described by Wawrzeńczyk J. and Kozak W. in their paper [12]. The sustainability requirements limit the spacing factor to 0.20 mm in Norway and Denmark and to 0.25 mm in Canada [13]. Yuan J. [14] experimented with the variation of concrete pores in the action of freeze–thaw cycles using X-ray tomography (CT). The results showed that there was no large variation in the amounts and volume of pores located in the range (0.5 mm^3^–20 mm^3^), but there were major changes for pores in another range. Tracz T. and Zdeb T. [15] studied the effects of hydration and carbonation on porosity and permeability at 90 days and up to 2 years of preservation on pastes made of ordinary Portland cement with different ratios of *w*/*c*. The results highlighted changes in capillary porosity caused by subsequent hydration advances and the carbonation phenomenon, while the depth of permeability did not change significantly during the monitored period.

Due to the pozzolanic and hydraulic properties, supplementary cementitious materials SCM may be used as a substitute for cement in the designed composition. They influence the properties of fresh concrete by their reaction with water or calcium hydroxide CH and forms additional hydrating compounds. Supplementary cementitious type materials may have pozzolanic properties by chemically reacting with calcium hydroxide to form additional hydrated calcium silicate C-S-H, which is a hydrating compound that has the greatest contribution to the development of concrete strength [6].

In this context the granulated and ground blast furnace slag GGBS in combination with ultrafine silica SF are used in the concrete industry for different applications because they have strength, durability, economic and environmental benefits.

Rao S.K. et al. [16] investigated the effect of GGBS on mechanical properties and the abrasion resistance of RCC for a level of substitution of cement with GGBS from 10% to 60%. Roller compacted concrete (RCC) is a stiff mixture of traditional concrete components that is proportioned with higher aggregate content and lower cementitious material content than compared to conventional concrete [6]. The test results showed that after the age of 28 days the increase in GGBS content led to an increase in the abrasion resistance of RCC at all replacement levels. Aghaeipour A. et al. [17] investigated the effects of GGBS as a substitute for cement in different percentages (20%, 40% and 60%) regarding water absorption, permeability and freeze–thaw resistance in RCC, with applications in road infrastructures in mind. The results not only showed a reduction in water absorption but also in mechanical resistances of concretes with GGBS compared to those without GGBS content. The depth of water penetration under pressure was lower for substitution levels up to 40%, after which it was higher than the value obtained from the control mixture. The lowest losses of resistance after 300 freeze–thaw cycles were registered in mixture with 60% substitution of GGBS, which is lower than the control mixture. Limbachiya V. et al. [18] made concrete paving blocks using GGBS and silica fume SF to replace the ordinary Portland cement OPC. High content of SiO_2_ in SF and of CaO in GGBS increased the durability properties of concrete paving blocks, which allowed the successful replacement of cement up to 40%. However, it was observed that the levigation properties of the blocks with GGBS and SF decreased, but within acceptable limits regarding the soil protection at levigation. In addition to improving the mechanical properties, the reduction in cracks developed in concrete during the different stages of hardening and densification is just as important for the durability of road pavements. Crack growth restriction has been studied in the works [19,20] by using hybrid fibers, such as steel-polyvinyl-polypropylene-calcium carbonate CaCO_3_, in cementitious compositions. The research conducted by Lam M.N.-T. et al. [21,22] investigated the feasibility of using slag aggregate resulting from electric arc furnace (FEA) to partially replace natural coarse aggregates and fly ash to partially replace cement.

The investigation methods regarding the effect of the supplementary cementitious materials are varied and depends on the envisaged properties of the manufactured materials. Moreover, research aims to establish the links between microscopic and macroscopic properties [23]. Nedunuri S.S.S.A. et al. [24] used water saturation and mercury porosimetry to study the evolution of pore structure as a function of the hardening age of Portland cement and of those that are partially replaced with SCM, such as SF, GGBS and fly ash. Majhi R.K. et al. [25] determined the rate of water absorption by capillary pores using the sorptivity test on concrete with GGBS and aggregates recycled from concrete in its composition. The microstructural characteristics extracted from scanning electron microscopy and X-ray diffraction, confirmed the results obtained by such classical methods. Renato J. et al. [26] studied the porosity of concrete using two non-destructive methods, which are X-ray microtomography and digital scanning. The porosity results obtained through the non-destructive methods were compatible with those obtained through the standard mercury intrusion porosimetry MIP test. However, the study by Diamond S. [27] drew attention to the fact that the MIP method is inadequate for measuring the pore size due to the method’s inability to penetrate the C-S-H gel pores; however, it is rather suitable for assessing the apparent porosity. The analysis of the pore structure was performed in the study [28] using the fractal theory, which allows a quantitative evaluation based on the fractal size of the pores. By the fractal model proposed by Jin S. et al. [29], a regression relationship was obtained between the fractal size and the durability factor. The characteristic parameters of the pore structure of the recycled concrete with residual fibers were investigated in the study conducted by Zhou J. et al. [30] by using the mercury intrusion porosimetry test and fractal theory. The results showed that the pore structure was mainly influenced by the water–cement ratio. The morphology of the pores developed in the first 7 days of hardening of the concrete was analyzed in the paper [31] relative to two different compositions. The first is made of simple concrete (PC) and the second is a mixture of high-performance reinforced concrete (SFRC). The internal voids of the mixtures in combination with the geometric parameters were identified by computed tomography (CT) scanning and the data were analyzed using digital image processing software (DIP) [32].

Kowalczyk R.M. et al. [33] highlighted, via nuclear magnetic resonance NMR relaxometry technique, the mechanism of water–isopropanol exchange in cement pastes. The results showed that the isopropanol revealed the presence of capillary pores better than revealed in the experiments on samples saturated with water. On the other hand, isopropanol draws water out from the gel pores but does not replace it in the same amount. One of the non-destructive methods commonly used to assess internal cracks resulting from the freeze–thaw phenomenon is the method of measuring the ultrasonic pulse propagation time (UPTT) in compliance with the Technical Report CEN/TR 15177:2006 [34]. The increase in propagation time of the wave through concrete shows a greater number of voids and internal cracks.

The great advantage of the NMR techniques is that they are completely non-invasive and, in addition, they permit the study of materials without prior preparation of samples. In the present work, NMR relaxometry [5,8,35,36,37,38,39,40,41] is used, again, to determine the residual water within the pores of the material and the effects of the freeze–thaw cycles on the porous system. Using this technique, information can be obtained about the porous structure of concrete containing a mixture of cement paste and aggregates, which is similar to the approach of Wawrzeńczyk J. and Kozak W. [12] and based on the Philleo concept. The NRM investigations on the effects of the freeze–thaw cycles complement the results obtained by measuring the physical characteristics (density, water absorption and content of permeable pores) of hardened road concrete determined by standard methods. The porosity results obtained from this experiment were compared to the results obtained in a previous experiment [42] on the compressive strengths and the loss of compressive strength after 300 freeze–thaw cycles. The microstructure of the concrete (in this case the pore sizes and the distance between them) can be investigated and analyzed using Scanning Electron Microscopy (SEM). The porosity induced by freeze–thaw cycles was analyzed for three mixtures, prepared with blast furnace slag and compared with two conventional mixtures used in road pavements. The investigation methods aimed to obtain more accurate conclusions for the selection of the optimal mixture of the blast furnace slag that would ensure durability for road concrete.

## 2. Materials and Methods

### 2.1. Materials

The Portland cement used for preparing the samples is of the CEM I 42,5R type and it was supplied by Lafarge Holcim S.A. The performances stated by the manufacturer are in compliance with the technical specification from SR EN 197-1:2011 [43]. The specified cement surface is 4385 cm^2^/g and the specific weight is 3.00 g/cm^3^. The granulated blast furnace slag 0/12.5 mm obtained by sudden cooling in water was supplied by the company Liberty (Galați, Romania). After grounding to under 69 µm, the granulated and ground blast furnace slag GGBS reached the specific surface of 5770 cm^2^/g and specific weight of 2.77 g/cm^3^. The admissibility conditions of the GGBS blast furnace slag were in compliance with the technical specifications from SR EN 15167-1:2007 [44]. The high content of 95% vitreous mass and the 1.15 ratio between calcium oxide and silicon oxide demonstrates good activity. The value of the activity index at 28 days was 74.94%. The values obtained for the alkalis content of 1% and the 0% calcination loss show that the GGBS used does not generate expansion by its use in concrete mixtures. Table 1 demonstrates the oxide composition for cement and (GGBS).

The aggregate proportions used in the preparation approach were 32% sand (sand dimension of 0/4 mm) and 68% coarse aggregate with a maximum grain dimension of 25 mm. The selection of aggregates was in compliance with the national standard NE 014:2002 [45] for road coating with role of wear and the physical properties were in compliance with SR EN 12620:2003 and SR EN 12620+A1:2008 [46]. The granulometric curve of the total aggregate mixture followed the framing within the granulometric area that is permitted by the standard NE 014-2002 [45]. The coarse aggregates were made of crushed river gravel (4/8) and crushed quarry rock (dacite from the category of igneous rocks) that were (8/16) and (16/25) mm, respectively. The use of crushed aggregates enables better adhesion between the aggregate and the cement paste due to the irregular surface, which is beneficial for the resistance to bending. The crushed strength of 14% (expressed by the Los Angeles coefficient) and the wear resistance of the aggregates of 6% (expressed by the micro-Deval coefficient) demonstrate superior performance compared to the standard SR EN 12,620 [46] result of tests performed by the manufacturer. The artificial aggregate from ungranulated blast furnace slag (ACBFS) is a crystalline by-product resulting from the solidification in air of molten blast furnace with applications in road pavements [47]. The characteristics of natural sand and aggregates (ACBFS) were determined by the manufacturer and are presented in Table 2.

The additives used here are commercially available from Badische Anilin und Soda Fabrik (BASF, Ludwigshafen, Germany). As a superplasticizer, we used MasterGlenium SKY 527 and air training additive Master Air 9060 (BASF, Germany) and both possess characteristics in compliance with SR EN 934-2+A1:2012 [48]. The water in the concrete mixture was taken from the water supply system of the city Cluj-Napoca in compliance with SR EN 1008:2003 [49].

### 2.2. Sample Preparation

The parameters of designing the road concrete complied with the minimum requirements for the severe exposure class (XF4) in accordance with SR EN 206-1:2002 and SR EN 206+A1:2017 [50]. The classification in conditions of exposure according to the XF4 class requires a minimum cement dosage of 350 kg/m^3^, maximum *w*/*c* ratio of 0.45 and a minimum strength class of C35/45 with entrained air [50,51]. A number of five mixtures were prepared with different quantities of materials presented in Table 3. The first two control mixtures were prepared using Portland cement type CEM I 42,5R utilizing natural aggregates and the following three mixtures were prepared using granulated and crushed blast furnace slag, under 63 µm, and aggregates from crushed blast furnace slag at a dimension of 0/4 mm in different proportions. The below observations were used:

**S 360**, control mixture of 360 kg/m^3^ Portland cement dosage and natural aggregates;
**S 414**, control mixture of 414 kg/m^3^ Portland cement dosage and natural aggregates;
**S 54/20**, 360 kg/m^3^ (cement) + 54 kg/m^3^ (GGBS) and 20% (ACBFS)_0/4 mm + 80% (NA)_0/4;
**S 54/40**, 360 kg/m^3^ (cement) + 54 kg/m^3^ (GGBS) and 40% (ACBFS)_0/4 mm + 60% (NA)_0/4;
**S 54/60**, 360 kg/m^3^ (cement) + 54 kg/m^3^ (GGBS) and 60% (ACBFS)_0/4 mm + 40% (NA)_0/4.

Compared to the control mixture S 360, the blast furnace slag (GGBS) was brought as a contribution to the cement mass in a percentage of 15% and, compared to the control mixture S 414, the GGBS substituted the cement in a percentage of 13%. The ground granulated blast furnace slag was used in road concrete mixtures as a binder with supplementary constituents with cement characteristics similar to those described in the study [52]. Natural sand NA was substituted in percentages of 20%, 40% and 60% with crushed blast furnace slag aggregates ACBFS with dimensions of 0/4 mm. In this experiment the water content and additive content was adjusted to maintain the consistency of the concrete at the most appropriate values of 20 mm.

### 2.3. Methods

#### 2.3.1. Sampling, Preservation and Preparation of Samples for Testing

For each mixture, 8 cubic specimens with a side of 150 mm and 3 specimens with a side of 71 ± 1.5 mm, as shown in Table 4, were prepared and kept in the air for 24 h after which they were stripped and immersed in water at a temperature of 20 ± 2 °C.

**Figure 1:**
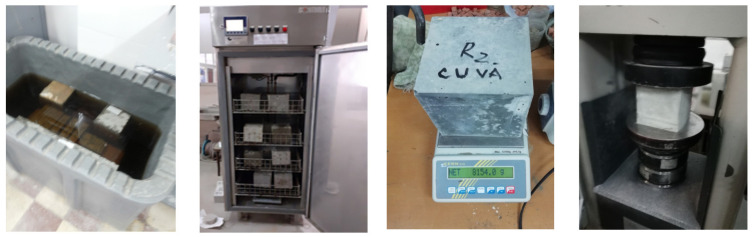
Control specimens kept in water and specimens underwent the freeze–thaw test (150 mm × 150 mm × 150 mm).

At the age of 7 days, all specimens were removed from water and stored in the climatic chamber at a temperature of 20 ± 2 °C and at a humidity of 65% ± 5% up to the age of 50 days. Next, the cubic specimen with the side of 71 mm was kept in water until the age of 100 days and the abrasion resistance was then determined; the values of are found in paper [53]. From the age of 100 days to the age of 150 days they were kept in the air in the climate chamber. The density, water absorption and content of permeable pores were determined from the average of the results for the 3 cubic specimens for each type of mixture with the side of 71 mm and at the age of 150 days in compliance with the standard ASTM C642-2006 [54]. The nuclear magnetic resonance measurements were performed on concrete cores extracted from two intact 150 mm cubes for each mixture; one tested for 300 freeze–thaw cycles and one control specimen maintained in water until the age of 150 days. For the NMR measurements, the cubes with apparent density close to the average of the 3 cubes tested for the freeze–thaw in the study were selected [42] by the method of the loss of compressive strength according to SR 3518:2009 [55]. The thermostatic chamber was set to maintain a temperature of (−17 ± 2) °C for 4 h for the freeze cycle, up to (20 ± 2) °C for the thaw cycle for 4 h and the humidity of RH 95%. Four days before the commencement of the test, the specimens were placed in a water tank at a temperature of (20 ± 5) °C for saturation and the control specimens were kept in water. Those intended for the freeze–thaw cycles were introduced in the thermostatic chamber, such as in Figure 1.

After 300 repeated freeze–thaw cycles, the specimens were tested for compression and the compressive strength losses were calculated, for the specimens tested with freeze–thaw cycles compared to the control specimens, with Equation (1) as follows:

(1) $$\eta_{n}=\frac{f_{cmwater}-f_{cmfreez}}{f_{cmwater}}\times 100\;\left(\%\right)$$

where:

*η_n_*—the compressive strength loss after “*n*” freeze–thaw cycles;
*f _cm water_*—the average compressive strength of the samples maintained in water during “*n*” freeze–thaw cycles;
*f _cm freeze_*—the average compressive resistance of the samples maintained in the thermostat chamber during “*n*” freeze–thaw cycles.

Compressive strength at 150 days was determined by applying a uniform and continuous force increasing in increments of 0.5 MPa/s on a loading machine type Advantest 9 of 300 tf. in accordance with SR EN 12390-3:2002 [56] and is calculated as by Equation (2) as follows:

(2) $$f_{c}=\frac{F}{A_{c}}\;\left(\mathrm{MPa}\right)$$

where:

*f_c_*—compressive strength in N/mm^2^,
*F*—the maximum load in N;
*A_c_*—cross-sectional area of the section of rupture in mm^2^.

#### 2.3.2. NMR Relaxometry Approach for Determining the Pore Size Distribution

The NMR relaxometry techniques are completely non-invasive and allow the investigation of cement samples without special prior preparation. These have been used successfully in the recent years to extract information about the porous structure of cement-based materials and to extract information with respect to the evolution of water inside the pores during the hydration process [5,8,35,36,37,38,39,40]. In the NMR relaxometry of cement-based materials, both the longitudinal relaxation time and the transverse relaxation time can be monitored [5,8,35,36,37,38,39,40]. Due to the fact that longitudinal relaxation measurements are slower and are more difficult to apply to the systems that evolve during the measurement process, it is preferable for many applications to measure the transverse relaxation time. However, in the case of transverse relaxation measurements, it is important to take into account the effects that diffusion in internal gradients could have on the accuracy of the measurement and so it is preferable that they be performed in low magnetic fields and with pulse sequences that could minimize internal gradients effects [5,8,40].

A technique often used in NMR applications on cement-based materials is known as the Carr-Purcell-Meiboom-Gill (CPMG) technique [57]. This technique allows the rapid and robust measurement of the transverse relaxation rate (1/T_2_) of the nuclear spins and if the time interval between radio frequency pulses is short, the influence of internal gradients can be neglected. Under these conditions, there is a relationship [5,8,37,38,39] between the transverse relaxation rate and the surface-volume (*S*/*V*) ratio of the pores that is described as follows.

(3) $$\frac{1}{T_{2}}=\varepsilon \frac{S}{V}$$

In the above equation, ε represents the surface relaxivity, which is a constant that depends on the interaction of molecules with the surface, the intensity of the magnetic field in which the NMR experiment is performed and the content of magnetic impurities of the pore surface. Note that in Equation (3) we neglected the contribution of bulk relaxation to the phenomenon of nuclear relaxation because the bulk relaxation time is much longer than the one induced by the surface. Based on Equation (2) we can establish a direct proportionality between the relaxation time and the pore size. This proportionality allows us to discover the distribution of pore dimensions if we know the distribution of relaxation times and the relaxivity of the surface. Even if the relaxivity of the surface is not known, information can be obtained about the relative distribution of pore sizes. This distribution can be extracted from the CPMG echo series if a numeric Laplacian transformation is applied to this series [58,59].

**Figure 2:**
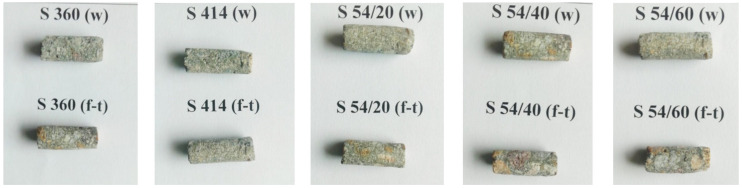
Samples ready for the NMR measurements.

In the present work, in order to highlight the effect of the freeze–thaw cycles on the relative distribution of pore sizes, the specimens were investigated by NMR relaxometry at the age of 150 days and after the completion of the 300 freeze–thaw cycles. The test period in the study was extended from the requirements of national standard NE014-2002 [45] from 100 to 300 freeze–thaw cycles and is similar to the number of freeze–thaw cycles provided by the standard ASTM C666/C666M-03 [60]. A laboratory exposure of concrete samples to an extended number of repeated freeze–thaw cycles can simulate, as closely as possible, the damage caused by the freeze–thaw phenomenon in concrete exposed to real weather, as experienced in the literature [61], where the samples were tested in a laboratory for up to 1000 freeze–thaw cycles. From the core of intact cubes kept in the thermostatic chamber and the control cubes kept in water during this period, cylindrical samples with a length of 20 mm and a diameter of 9.5 mm were extracted, such as in Figure 2. To eliminate interpretation errors, the mass of the samples extracted from the cubes was the same for each mixture of 1.06 g. It was possible to take samples for testing at these small sizes because the proportion of maximum size aggregates has a relatively small volume of the total volume of aggregates. The samples were dried in an oven at 105 ± 5 °C for 24 h to eliminate water from the pores. They were then inserted into 10 mm NMR tubes which were sealed to prevent molecular exchange with water from atmospheric air. After measuring the dry samples, they were dried again and immersed in water for 48 h at 22 ± 2 °C. After this interval, the samples were lightly buffered with filter paper, then inserted into the NMR tubes and sealed. The third set of measurements was performed on samples saturated with cyclohexane and follows the same procedure as in the case of samples saturated with water. The saturation with cyclohexane was performed to better highlight the inter-C-S-H pores and capillaries, as is demonstrated in the other study [8]. Note that the intra-C-S-H pores are highlighted by the presence of water molecules that cannot be removed without destroying the material [8].

The NMR measurements were performed with a low field instrument MinispecMQ20 (Bruker, Karlsruhe Germany) using the CPMG technique. Before each measurement, the samples were permitted to reach the thermal equilibrium at a temperature of 35 °C. A number of 2000 spin echoes were recorded in each experiment and the time between two echoes was maintained at 0.1 ms to reduce the effects of internal gradients on the measurements. Relaxation time distributions were extracted from the CPMG echo series using a Laplace numerical inversion [59].

#### 2.3.3. Scanning Electron Microscopy (SEM)

Small pieces of samples were taken from the interior part of the crushed samples and analyzed at room temperature using a VEGA3 SBU electronic microscope with an Energy Dispersive Spectrometer Quantax EDS from Bruker in order to obtain information about the pore’s sizes and the distance between the pores. Fragments from the 150 mm side cubes remaining after the NMR measurements were used from the control sample set, which was kept in water during the freeze–thaw test.

#### 2.3.4. Density after Immersion and Boiling, Water Absorption and Proportion of Permeable Pores

**Figure 3:**
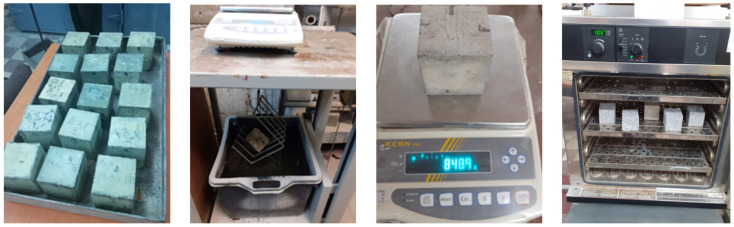
Hydrostatic weighing of cubes with 71 mm side.

The density after immersion and boiling, water absorption and permeable pore content were obtained by a method in accordance with ASTM C 642-2006 [54] that was used in another study [62]. The water-saturated samples with the dry saturated surface were weighted in air (b) and then dried in an oven at 100–110 °C until a constant mass (*a*) was reached. The samples were then placed in a suitable container, covered with tap water and boiled for 5 h. They were then allowed to cool to room temperature (22 ± 2 °C). The moisture from the surface was removed with a towel and the dry mass of the surface-dry sample suspended in air after immersion and boiling was determined (*c*), with the mass measured in air. The apparent mass (*d*), after immersion and boiling, was measured in water with the hydrostatic balance, such as in Figure 3. The absorption after immersion (*m*_1_) and absorption after immersion and boiling (*m*_2_) were calculated with the following equations.

(4) $$m_{1}\;=\;\left[\left(b-a\right)/a\right]\;\times \;100$$

(5) $$m_{2}\;=\;\left[\left(c-a\right)/a\right]\;\times \;100$$

Dry mass density, *ρ*_1_, and density after saturation and boiling, *ρ*_2_, were calculated with the following equations:

(6) $$\rho_{1}\;=\;\left[a/\left(c-d\right)\right]\;\times \;\rho_{w}$$

and

(7) $$\rho_{2}\;=\;\left[a/\left(a-d\right)\right]\;\times \;\rho_{w}$$

For water density, *ρw*, the value of 0.998 g/cm^3^ was used. The permeable pore proportion was calculated with the equation:

(8) $$P_{0}\;=\;\left[\left(\rho_{2}-\rho_{1}\right)/\rho_{1}\right]\;\times \;100,\;\mathrm{specified}\;\mathrm{in}\;\%$$

which is in accordance with ASTM C 642-2006 [54] standard.

## 3. Results and Discussion

### 3.1. Relative Distribution of Pore Sizes

**Figure 4:**
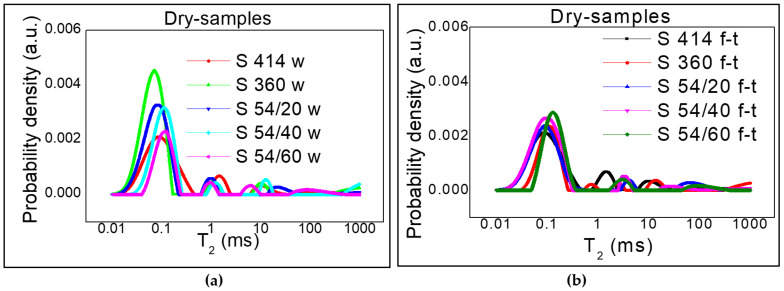
Distribution of the transverse relaxation time T_2_ after drying: (**a**) control samples kept in water (w); (**b**) samples subjected to 300 freeze–thaw cycles (f-t).

**Figure 5:**
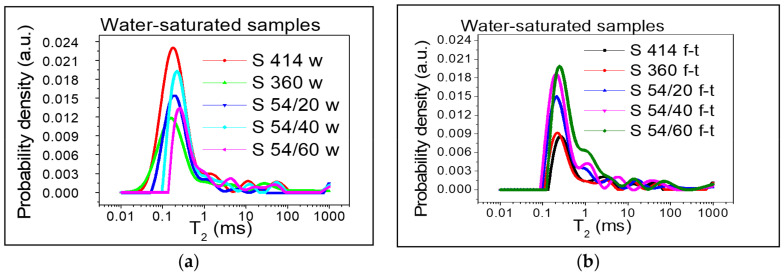
Distribution of the transverse relaxation time T_2_ after water saturation: (**a**) control samples kept in water (w); (**b**) samples subjected to 300 freeze–thaw cycles (f-t).

The distribution of transverse relaxation times for the gel pores obtained from the Laplace numerical inversion, for each composition described in Table 3, is shown in Figure 4 and Figure 5. Three peaks can be distinguished in all cases and the position of the peaks can be associated with the three types of pores. The values recorded on the horizontal axis for the transverse relaxation time T_2_ are proportional to the pore size (see Equation (3)) and the values recorded on the vertical axis show the probability of having a certain pore dimension. The measuring unit on the y-axis is arbitrary (a.u. = arbitrary units). For a direct comparison of the curves, the same scale was used at each measurement performed.

Figure 4 depicts the samples dried according to the procedure described above, both control samples (Figure 4a) and those subjected to the freeze–thaw process (Figure 4b). A small amount of water (the first peak) was observed in the gel-like pores (intra C-S-H) with dimensions up to 2 nm [8], which could not be removed during drying process. In the mixture S 360, the distribution of transverse relaxation times corresponding to the gel pores (intra C-S-H) for the control samples, preserved in water (Figure 4a), shows a maximum placed in the range 0.02–0.3 ms and indicated a maximum probability density of 0.0045 for a value of T_2_ of 0.08 ms. At T_2_, the values slightly shifted to the right and the maximum probability density decreased to 0.003 for the mixtures S 54/20 and S 54/40 followed by the mixtures S 414 and S 54/60, with an intensity inferior to 0.002. However, for the samples subjected to 300 freeze–thaw cycles (Figure 5b), the transverse relaxation time distribution interval shifted to the right (for S 414, S 54/20, S 54/40) at the higher pore size values between 0.02–0.6 ms and the most accentuated values were recorded in the mixtures S 360 and S 54/60 between 0.06–0.6. For all mixtures stored in the refrigerator (f-t), a maximum probability density close to the value recorded for the control mixtures, 0.003, was registered. The results of the experiment show that, after the freeze–thaw test, the pore size was higher compared to the control mixtures, which indicated the appearance of microcracks even in the intra C-S-H pores.

By introducing the samples into the water, the clear increase in the area of the peaks corresponding to the gel pores (intra C-S-H and inter C-S-H) can be observed in Figure 5.

**Figure 6:**
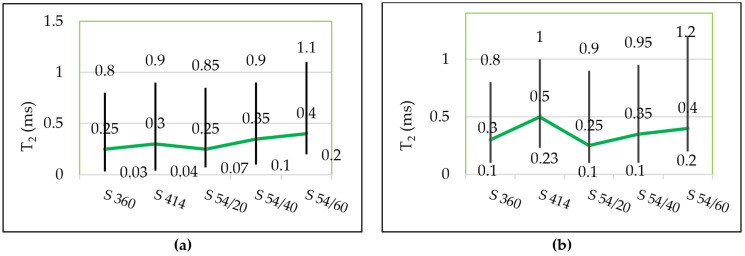
The distribution range of the gel pores for: (**a**) the control samples; (**b**) for samples subjected to freeze–thaw cycles. Both measurements are performed on water-saturated samples.

The pore distribution continues with several lower intensity peaks, which correspond to capillary pores, cracks or are artefacts of the numerical inverse Laplace. In the control samples (Figure 5a) the distribution of the transverse relaxation times was within the range 0.03–1.10 ms and for the mixtures tested for freeze–thaw (Figure 5b) the peak shift to the right was recorded at higher values, in the range 0.1–1.20 ms, as can be observed in Figure 6, which shows the starting and ending values for T_2_ in each mixture corresponding to the gel pores as well as the position of the maximum (a.u.) (broken line).

The relative distribution of pore sizes in mixture S 54/20 and S 54/40 was kept close to the distribution in mixture S 360 and lower than the distribution in the control mixture S414 in both control samples and the samples tested for freeze–thaw. However, for the 54/60 mixture, the distribution range shifted to higher values compared to the two reference mixtures in both the control specimens and in the specimens that underwent the freeze–thaw text.

**Figure 7:**
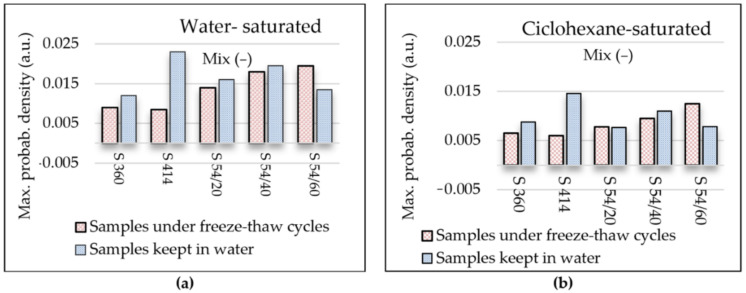
Maximum value of the probability density corresponding to the gel pores in the case of water saturated (**a**) and cyclohexane saturated (**b**) samples.

Figure 7a shows the maximum probability density recorded in both control samples kept in water (w) and in samples tested in water-saturated freeze–thaw (f-t). For the water-saturated control samples (Figure 7a), the maximum probability density with the highest value was registered in the mixture S 414 (of 0.023) and the lowest value (of 0.012) was obtained in the mixture S 360. In all blast furnace slag mixtures, the maximum probability density value corresponding to the gel pores is higher than the reference mixture S 360, but lower than in the control mixture S 414. For all water-saturated samples tested at 300 freeze–thaw cycles (Figure 7a), it was observed that the maximum value of the probability density corresponding to the peak of the gel pores increases with the increase in the percentage of substitution with blast furnace slag aggregate and it is above the level in the control mixtures S 360 and S 414. The largest difference in the maximum value of the probability density compared to the control sample was recorded in the mixture S 54/60 maintained at freeze–thaw.

**Figure 8:**
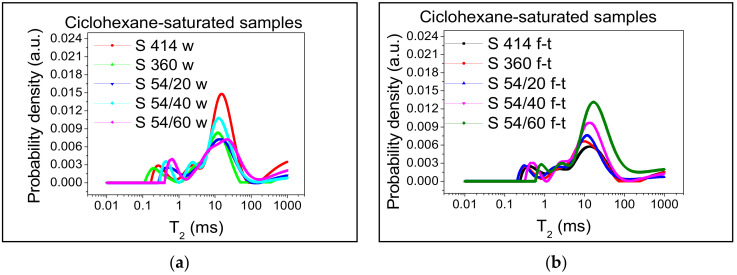
Transverse relaxation time distribution T_2_ after cyclohexane saturation: (**a**) control samples kept in water (w); (**b**) samples subject to 300 freeze–thaw cycles (f-t).

For the better monitoring of the capillary pores, it is preferable that they are saturated with cyclohexane because a higher degree of filling can be obtained in the case of cyclohexane than in the case of water [8]. This is due to the significantly lower contact angles in the case of cyclohexane than water relative to the mineral surfaces inside the cement paste [8,63]. Figure 8 shows the distribution of relaxation times and Figure 7b shows the maximum probability density corresponding to the peak of the capillary pores in the samples saturated with cyclohexane.

**Figure 9:**
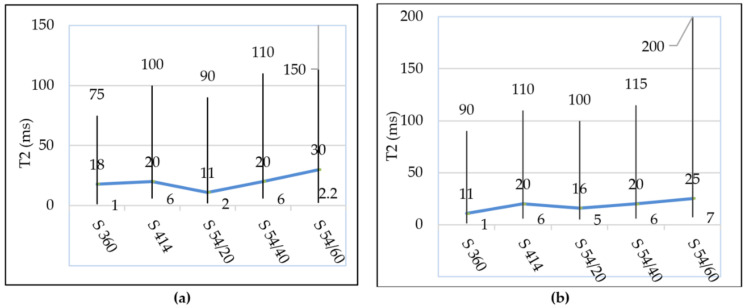
The capillary pores distribution range: (**a**) for the control samples (w); (**b**) for the samples tested in freeze–thaw (f-t). Both measurements were made on cyclohexane-saturated samples.

It is observed in Figure 7b that the evolution of the maximum probability density of the capillary pores has a tendency similar to that of the gel pores from the water-filled samples (Figure 7a); in all compositions with blast furnace slag, the intensity of the capillary pores increases with the increasing level of substitution of slag aggregate, respectively. In the control samples, the shortest range of distribution of capillary pores was recorded in the mixture S 360 situated between 1.0–75 ms and the largest range was recorded in the mixture S 54/60 between 2.2–150 ms, according to Figure 9a.

In samples subjected to 300 freeze–thaw cycles, the transverse relaxation time distribution T_2_ is slightly shifted to the right in mixture S 360 between 1.0–90 ms and more accentuated in mixture S 54/60 between 7.0–200 ms, as can be seen in Figure 9b. In the S 54/60 mixture, the peak corresponding to the capillary pores does not tend towards the minimum intensity in the position of T_2_ (200 ms), which indicated the presence of the larger capillary pores or internal cracks in concrete.

A longer relaxation time (Figure 9b) and a higher intensity (Figure 9b) in the S 54/60 mixture show an increase in the size and the proportion of capillary pores in the samples tested at freeze–thaw (f-t) compared to the control specimens kept in water (w).

This change in the dimension of capillary pores from the samples subjected to freeze–thaw (f-t) is explained by the fact that the phenomenon of frost water enters the structure of concrete capillary pores, freezes at about −0.5 °C and the resulting volume of the ice formed is approximately 9% higher compared to the water initially absorbed [1,2,3]. Thus, repeated freeze–thaw cycles lead to an increase in the size of the capillary pores and the appearance of cracks.

### 3.2. Pores Size

**Figure 10:**
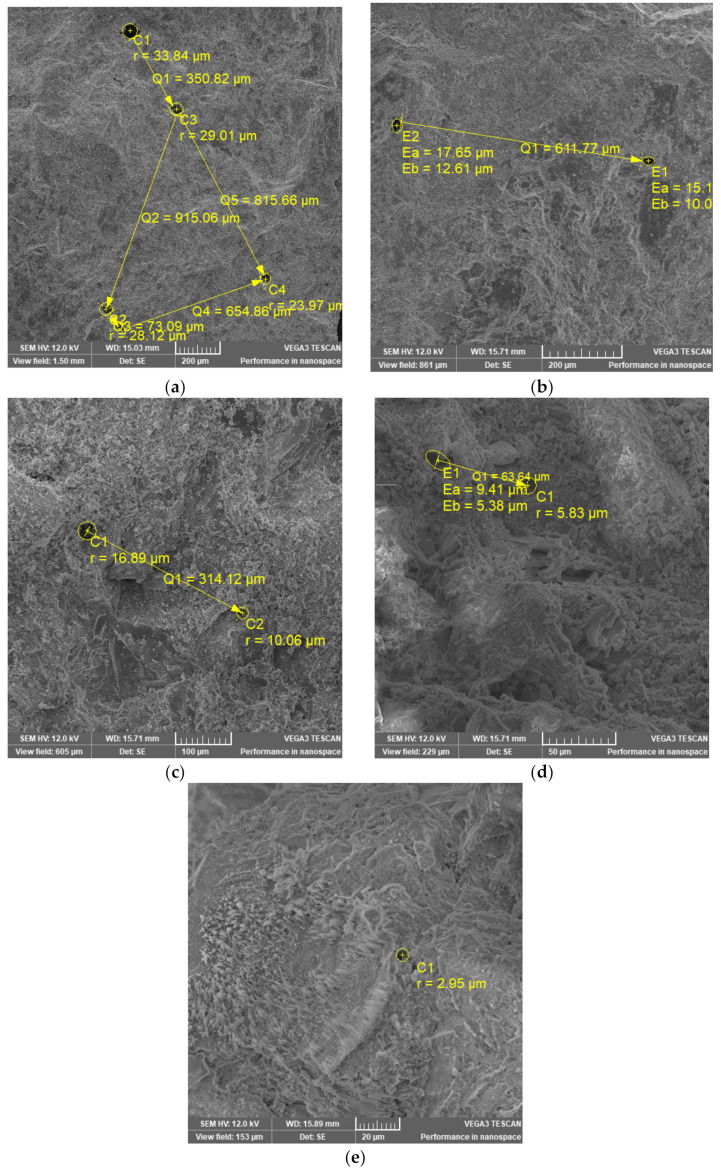
SEM images of S360 (**a**), S414 (**b**), S54/20 (**c**), S54/40 (**d**) and S54/60 (**e**).

SEM images obtained for the investigated samples are presented in Figure 10. In order to obtain a better view of the pores, all samples were measured using the same parameters but at different magnifications. The pores radius and the distance between pores were measured automatically by SEM software after selecting the shape most appropriate (circle or ellipse) to the shape of the pores. It can be observed that the radius of the pores ranges from ~33.84 μm for S 360 to ~2.95 μm for S 54/60. The distance between pores ranges from ~915.06 μm for S360 to ~63.64 μm for S 54/40. For S 54/60, no pores were found in the close vicinity of the selected pore to measure the distance. Pore radii with dimensions over ~2.95 μm indicate their framing at capillary pores in accordance with Reference [10]. The results obtained for the S 360 mixture suggest a lower pore density because the largest pore distance was identified. These results can be correlated with those obtained from the technique (NMR), which identified the lowest density of capillary pores on the control sample S 360 (Figure 7a). For the mixture S 54/20 and S 54/40 the distance between the pores was shorter compared to the mixture S 360, which suggests a higher pore density. It is observed that the measurements (NMR) show the same increase in the probability density in the compositions S 54/20 and S 54/40 compared to the mixture S 360 (Figure 7a).

### 3.3. Density, Water Absorption and Permeable Pores Content of Hardened Concrete

**Figure 11:**
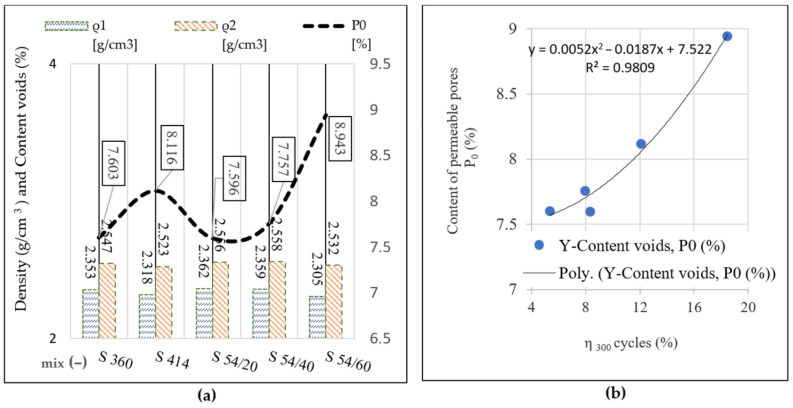
(**a**) Dry density (ρ_1_), density after immersion and boiling (ρ_2_) and content of permeable pores (P_0_) at the age of 150 days; (**b**) the relationship between the loss of compressive strength after 300 freeze–thaw cycles (η_300_) and the permeable pore content (P_0_) of the control samples.

Following the determinations performed in compliance with [54] on cubes with side 71 mm, the proportion of permeable pores (P_0_), dry density (ρ_1_) and density after saturation and boiling (ρ_2_) are illustrated in Figure 11a, respectively. The reported results represent the average of three samples tested in each mixture and the standard deviation (SD) and the coefficient of variation (CoV) are shown in Table 5.

Evaluation of compressive strengths and loss of compressive strengths (η_300_) after 300 freeze–thaw cycles determined on cubes with a 150 mm side, according to SR 3518:2009 [55], are found in the other studies [42] and included in Table 6. The compressive strengths for the control samples and for the samples tested at 300 freeze–thaw cycles recorded a standard deviation (SD) in the range (0.15–5.53) MPa and the coefficient of variation (CoV) between (0.2–9.1) %. It is observed that the scattering of results is below the accepted limit of 15%, while having a reasonable quality in the range of 5% to 10% as suggested in the study [64].

Analyzing the results from Table 6, it is observed that the compressive strengths determined on the control specimens have a similar evolution with the results of the densities presented in Figure 11a. Furthermore, the value of compressive strength losses after 300 freeze–thaw cycles tends to evolve similarly to that of the permeable pore content and develops a second order polynomial relation having a very good correlation coefficient (R value), shown in the diagram in Figure 11b.

**Figure 12:**
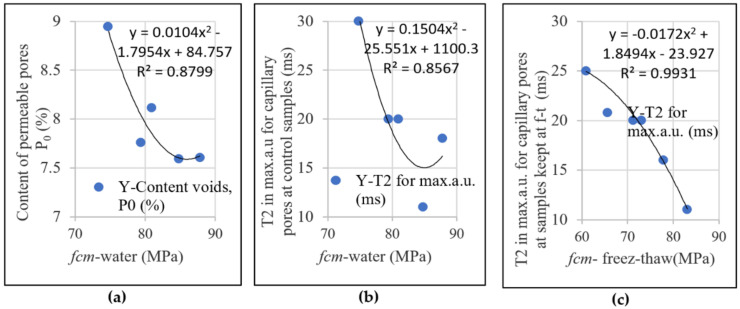
Relationship between compressive strength (*fcm*) and porosity: (**a**) relative to the permeable pore content (P_0_) in the control samples; (**b**) relative to the relative pore size for the position in which the transverse relaxation time (T_2_) records the maximum density in the control samples; (**c**) relative to (T_2_) for the position in which it records the maximum density at the samples kept at freeze–thaw (f-t).

In Figure 12, it is observed that the compressive strengths determined on the control specimens and on specimens maintained at 300 freeze–thaw cycles maintain their evolution trend with the content of permeable pores (Figure 12a) with the relative size of the capillary pores evaluated by NMR technique (Figure 12b,c). With the reduction in the content of permeable pores (P_0_) and the transverse relaxation time (T_2_), for which the probability density (au) recorded the maximum value (broken line in Figure 9a,b), the result is the increase in mechanical strengths. The polynomial relation of order two, derived by regression, developed between the compressive strength and the content of permeable pores and the relative size of the capillary pores determined on the control samples, possesses a value of the correlation coefficient very close to 0.8779 and 0.8567 (Figure 12a,b), which confirms the concordance results obtained by standard methods and NMR technique. Moreover, the confirmation of the results obtained by the NMR technique can be appreciated from the correlation coefficient 0.9931 (having the standard error of only 6.9 × 10^−3^) obtained from the polynomial relationship developed between the compressive strength and the transverse-room relaxation time (T_2_) for the maximum (a.u.) capillary pores and are measured on samples maintained at 300 freeze–thaw cycles (Figure 12c).

The permeable pore content obtained for the mixture S 54/20 is lower compared to the two control mixtures S 360 and S 414. The mixture S 54/40 is above the level of the control mixture S 360 and below the level of S 414. However, at mixture S 54/60, in which the substitution with artificial aggregates was 60% the porosity of the concrete increased the most, it was above the level of the two control mixtures. For blast furnace slag mixtures, the mechanical strengths decreased as the level of substitution with slag aggregates increased and the lowest values were registered for the S 54/60 mixture. The cause that led to the decrease in the mechanical strengths was the increase in capillary porosity, which in turn was influenced by the amount of water used and by the increase in water/binder ratio; this is a fact shown in the specialty literature [6]. The increase in the specific surface of the ground furnace blast slag and the increase in the porosity of the slag aggregate led to the increase in the water requirement in the preparation of the mixtures S 54/40 and S 54/60 in order to obtain a similar consistency to the reference mixtures; the results are in compliance with the specialty literature [65]. The crushed slag aggregates have an angular shape, which reduces the workability of the concrete and have a higher porosity than natural sand [47]. Similarly, the grinding fineness of the blast furnace slag was higher than that of cement; the specific surface in furnace blast slag was 5770 cm^2^/g and in cement it was 4385 cm^2^/g. However, for the mixture S 54/20, the proportions of blast furnace slag used led to the increase in the mechanical resistances and to the decrease in the porosity, obtaining better performances than the control mixture S 414 at the same water/binder ratio. For this mixture, the use of slag as a binder (GGBS) and aggregates (ACBFS) in low dosages was advantageous.

## 4. Conclusions

The connectivity of gel (intra C-S-H and inter C-S-H) and capillary pore network was investigated for the first time on road concrete samples tested for repeated freeze–thaw cycles through low field NMR relaxometry. The experiments rely on monitoring the distribution of water and cyclohexane molecules saturating the pores. The relative distribution of gel and capillary pores, determined through NMR, was conducted on samples taken from control specimens kept in water during the freeze–thaw test period and on samples extracted from specimens tested at 300 freeze–thaw cycles. The content of permeable pores was obtained on cubes with a side of 71 mm, made of the same composition and tested at the same age. For the three blast furnace slag compositions, the same amount of (GGBS), 54 kg/m^3^, but different percentages (20%, 40%, 60%) of crushed aggregates (ACFBS) were used. Different dosage of Portland cement, 360 and 414 kg/m^3^, were used for the two compositions made with conventional materials. The conclusions of the experiments are summarized in the following:

- After the freeze–thaw cycles, the transverse relaxation time distribution interval, T_2_, a of the intra C-S-H pores shifted towards higher values compared to the control mixtures, which indicates the appearance of microcracks even in the (intra C-S-H) pores. In addition, the maximum probability density was close to the value indicated for the control mixtures.
- From the diagrams of the distribution of inter C-S-H gel pores and of the capillary pores of the specimens tested at freeze–thaw, displacements are observed for higher values of T_2_ in all compositions with blast furnace slag compared to the control samples, which indicates an increase in the relative size of the pores.
- Furthermore, the maximum probability density of samples maintained at freeze–thaw cycles, measured on the y-axis, increased with the increase in the substitution level of crushed aggregates, indicating the increase in the density of the pore distribution in concrete with the highest increase registered in the S 54/60 mixture.
- For the S 54/40 and S 54/60 mixtures tested on freeze–thaw, the distribution range of the capillary pores was shifted to higher values compared to the control mixtures, which showed an increase in pore size due to the increase in the amount of water in the capillary pore mixtures.
- However, for the mixture S 54/20, the distribution range and the maximum intensity of the capillary pores were close to the control mixture S 360 and lower than the control mixture S 414, which indicates that this mixture contains the optimal dosages of slag built-in furnace.
- The results obtained for the content of permeable pores and the mechanical strengths obtained through standard methods and of capillary pore distribution obtained through NMR technique were consistent.
- The analysis of SEM images for slag mixtures shows that the pore density for S 54/20 mixture is the lowest, which confirms the highest compressive strength.
- Using the CPMG technique, the distribution and relative size of gel pores and capillaries on concrete samples tested on freeze–thaw were revealed, which permits the additional extraction of gel pore information compared to the standard method in which only the content of permeable pores is extracted.
- The CPMG technique can reflect the effect of freeze–thaw cycles on the total porosity of the concrete internal structure; however, the results must be correlated with standardized methods.
- An important advantage of this technique is that it allows the progressive and repeated evaluation of the distribution of gel pores and capillaries throughout the freeze–thaw test.

## Figures and Tables

**Table 1:** Oxide content for cement CEM I 42.5R and (GGBS) (%).

Oxides	SiO_2_	Al_2_O_3_	MnO	MgO	CaO	Fe_2_O_3_	Na_2_O	K_2_O
CEM I 42.5R	18.57	3.09	3.72	0.70	63.93	4.84	0.12	0.74
GGBS	36.44	11.60	0.55	5.8	41.81	0.78	0.345	0.428

**Table 2:** Characteristics of natural sand (NA) and of aggregates from blast furnace slag (ACBFS), 0/4 mm.

Technical Characteristics	Obtained Values ACBSF_0/4 mm	Obtained Values NA_0/4 mm	Limits SR EN 12620
Granularity	GF _85_	GF _85_	GF _85_
Coefficient of water absorption	WA_24_2	WA242	-
Content of fine particles <0.063 mm, %	f_3.5_	f_3_	(3 ÷ 22)
Sulphate soluble in acid, %	AS _0.52_	-	≤1.0
Total sulphate, %	0.96	-	≤2.0
Disintegration of iron from blast furnace slag	Does not present cracks and disintegrate	-	Visual aspect
Disintegration of dicalcium silicate from blast furnace slag	Presents a uniform violet color, with shining stains in small quantities uniformly distributed	-	Visual aspect

**Table 3:** Material quantities in mixtures.

Quantities (kg/m^3^)	Mixtures (kg/m^3^)				
	S 360	S 414	S 54/20	S 54/40	S 54/60
Cement (C)	360	414	360	360	360
Blast furnace slag powder (GGBS)	-	-	54	54	54
Total binder (L)	360	414	414	414	414
Water (W)	156.60	169.74	167.67	178.02	178.02
W/L, (water/binder)	0.44	0.41	0.41	0.43	0.43
Natural sand (NA 0/4 mm)	596	586	477	347	232
Blast furnace slag aggregate (ACBFS 0/4 mm)	-	-	119	232	347
Coarse aggregate (CA 4/25 mm)	1268	1245	1267	1231	1231
Total aggregate	1864	1831	1863	1810	1810
Superplasticizer additive	3.60	4.14	4.14	4.97	4.97
Air training additive	1.08	2.07	2.07	2.07	2.07
Slump (mm)	14	15	13	16	15
Fresh state density	2380	2415	2444	2402	2402

**Table 4:** Number of samples prepared for testing in each composition.

Name of Tests (Test Method)	Number of Samples (Pieces)	Dimension (mm)	Trial Age (Days)
Dry density, density after immersion and boiling and content of permeable pores (ASTM C642: 2006)	3	71 mm	150
Control samples kept in water (w) (SR 3518: 2009)	3	150 mm	150
Tests tested at 300 cycles (f-t) (SR 3518: 2009)	3	150 mm	150
Control samples kept in water (w) (NMR relaxometry and SEM)	1	150 mm	150
Tests tested at 300 cycles (f-t) (NMR relaxometry)	1	150 mm	150

**Table 5:** Standard deviation (SD) and coefficient of variation (CoV) calculated for density and permeable pore content.

Mixture	S 360	S 414	S 54/20	S 54/40	S 54/60
SD-ρ1 (g/cm^3^)	0.005	0.019	0.007	0.001	0.013
CoV (%)	0.002	0.008	0.003	0.000	0.006
SD-ρ2 (g/cm^3^)	0.003	0.041	0.009	0.019	0.033
CoV (%)	0.001	0.016	0.004	0.008	0.013
SD-P_0_ (g/cm^3^)	0.195	1.425	0.763	0.730	1.717
CoV (%)	0.026	0.176	0.100	0.094	0.192

**Table 6:** The average compressive strengths, resistance losses after 300 freeze–thaw cycles [42], standard deviation and coefficient of variation.

Mixture	S 360	S 414	S 54/20	S 54/40	S 54/60
fcm—water (MPa)	87.78	80.96	84.80	79.38	74.77
SD (MPa)	4.802	1.078	0.855	1.528	4.748
CoV (%)	0.055	1.300	0.010	0.019	0.064
fcm—300 cycles (f-t) (MPa)	83.06	71.15	77.82	73.06	60.95
SD (MPa)	0.492	2.630	0.147	4.040	5.525
CoV (%)	0.006	0.037	0.002	0.055	0.091
Reduction in compressive strength (η_300_)	5.38	12.10	8.32	7.96	18.47
SD (MPa)	4.633	2.078	1.089	3.325	2.220
CoV (%)	0.862	0.171	0.132	0.415	0.120

## Data Availability

The data presented in this study are available on request from the corresponding author.
